# Mutational Profiling Detection in FNAC Samples of Different Types of Thyroid Neoplasms Using Targeted NGS

**DOI:** 10.3390/cancers17152429

**Published:** 2025-07-23

**Authors:** Riying Liang, Man Luo, Xinhua Yang, Baoming Luo, Rongbin Liu

**Affiliations:** 1 Department of Ultrasound, Sun Yat-sen Memorial Hospital, Sun Yat-sen University, Guangzhou 510120, China; liangry8@mail.sysu.edu.cn (R.L.); luom63@mail2.sysu.edu.cn (M.L.); 2 Guangdong Provincial Key Laboratory of Malignant Tumor Epigenetics and Gene Regulation, Sun Yat-sen Memorial Hospital, Sun Yat-sen University, Guangzhou 510120, China; 3 Department of Molecular Diagnostics, Sun Yat-sen University Cancer Center, Sun Yat-sen University, Guangzhou 510120, China; yangxh@sysucc.org.cn; 4 State Key Laboratory of Oncology in South China, Collaborative Innovation Center for Cancer Medicine, Guangzhou 510060, China

**Keywords:** thyroid neoplasms, molecular profiling, fine-needle aspiration cytology, next-generation sequencing

## Abstract

Background: Thyroid neoplasms exhibit a diverse molecular landscape, and the 2022 WHO classification emphasizes the critical role of molecular profiling in thyroid cancer management; however, comprehensive mutational data from fine-needle aspiration cytology (FNAC) samples using targeted next-generation sequencing (NGS) are still limited, necessitating further investigation to guide clinical practice. Purpose: To characterize the mutational landscape of thyroid neoplasms using targeted NGS of FNAC samples and to assess the clinical implications of molecular profiling. Materials and Methods: This retrospective study included 952 patients with thyroid carcinomaneoplasms who underwent surgery at Sun Yat-sen Memorial Hospital from 2021 to 2023. Preoperative ultrasound, FNAC, and targeted NGS were performed. NGS panels covering 18, 88, and pan-cancer genes were used to analyze FNAC samples. Molecular alterations were correlated with clinical and pathological features. Results: The most frequent mutation was BRAF^V600E^ (84.45%), followed by RET (6.41%), BRCA1/2 (4.41%) and RAS (4.41%). Patients were categorized into BRAF-like (830 cases), RAS-like (36 cases), high-risk mutations (25 cases), and other mutations (28 cases). High-risk mutations were associated with older age and larger tumor size. BRAF-like tumors had a higher lymph node metastasis rate (58.77%) compared to RAS-like tumors (33.33%). Tumor mutation burden varied significantly among different thyroid neoplasm subtypes. Conclusions: Molecular profiling using targeted NGS of FNAC samples provides valuable insights into the genetic landscape of thyroid neoplasms and has significant clinical implications for diagnosis and personalized treatment strategies. Further validation with paired tumor and plasma samples is warranted.

## Simple Summary

This retrospective study analyzed 952 patients with thyroid neoplasms using targeted next-generation sequencing (NGS) of fine-needle aspiration cytology (FNAC) samples. The most frequent mutation was BRAF^V600E^ (84.45%). High-risk mutations were associated with larger tumor size (*p* < 0.001) and older age (*p* < 0.001). BRAF-like tumors had a higher lymph node metastasis rate compared to RAS-like tumors (58.77% vs. 33.33%, *p* < 0.001). Our study identified BRAF^V600E^ as the most frequent mutation in thyroid neoplasms and revealed significant associations between molecular profiles, tumor size, and lymph node metastasis.

## 1. Introduction

Thyroid neoplasms represent the most prevalent class of endocrine tumors, predominantly manifesting as benign or malignant lesions arising from the thyroid gland [1]. The incidence of these tumors exhibits a female predilection, with a 2–4-fold higher occurrence in females compared to males [2,3]. Within the spectrum of benign tumors, thyroid adenomas are the most frequently observed, while papillary thyroid carcinomas constitute the majority of malignant tumors [4,5]. These neoplasms predominantly arise from the follicular epithelial cells of the thyroid gland. The 2022 World Health Organization (WHO) classification system for thyroid neoplasms delineates follicular cell-derived tumors into three principal categories based on criteria including cellular origin, pathological characteristics, molecular profiling, and biological behavior [6]. According to the new classification, thyroid neoplasms (TN) are divided into benign tumors (BT), low-risk neoplasms (LRN), and malignant neoplasms. BTs include not only follicular adenomas but also significant variants, such as those with papillary architecture. LRNs includes NIFTP, thyroid tumors of uncertain malignant potential, and hyalinizing trabecular tumors. Malignant tumors include differentiated high-grade thyroid carcinoma (DHGTC), poorly differentiated thyroid carcinoma, PDTC), anaplastic thyroid carcinoma and differentiated thyroid carcinoma (DTC) which includes papillary thyroid carcinoma (PTC), follicular thyroid carcinoma (FTC), and oncocytic carcinoma of the thyroid carcinoma [6].The 2022 WHO classification underscores the pivotal role of driver genes in the etiology and progression of thyroid cancer [7]. Identification of mutations in genes such as BRAF^V600E^, TERT promoter, RAS, and RET fusions furnishes robust evidence for adjunctive diagnosis, elucidation of tumor biology, prognostic stratification, and the implementation of targeted therapy for patients afflicted with advanced PTC, high-grade thyroid follicular epithelium-derived neoplasms, and undifferentiated carcinomas [8,9].

Ultrasound-guided fine-needle aspiration biopsy has emerged as a critical diagnostic modality for discerning the benign or malignant nature of thyroid neoplasms in clinical practice [10], circumventing the need for surgical excision and consequent patient discomfort [11]. Molecular interrogation of genetic aberrations within thyroid cancer has the potential to augment the diagnostic fidelity of cytological assessments and to refine the prognostication of clinical outcomes [12,13]. The detection of specific molecular markers, including BRAF^V600E^, RAS, and RET fusions, in FNA samples is instrumental in significantly amplifying the diagnostic accuracy [14]. Preoperative assessment of BRAF^V600E^ mutation status is also of paramount importance in the diagnosis and prognostic prediction of PTC, expediting the formulation of personalized treatment regimens. Consequently, multigene analysis of cytological specimens allows clinicians to garner a more holistic understanding of patients’ conditions, thereby informing the selection of appropriate surgical intervention strategies.

NGS, a cost-effective and high-throughput methodology, identifies clinically actionable mutations across a spectrum of genes [15]. NGS-based multi-gene panel testing has markedly enhanced the diagnostic precision of FNA [16]. For thyroid neoplasms with indeterminate malignancy by FNA, the assessment of specific molecular markers such as BRAF^V600E^, RAS, and RET fusions in cytological samples can refine diagnostic accuracy. The detection of BRAF^V600E^ mutation in FNA cytological samples is strongly indicative of malignancy, while RET fusions are highly specific for the diagnosis of papillary thyroid carcinoma [17]. Although RAS mutations alone do not confer a high risk of malignancy in thyroid nodules [18], their co-occurrence with other mutations, particularly within the TERT promoter, EIF1AX, or TP53 genes, significantly amplifies the risk of aggressive thyroid cancer [19]. Genetic variations, including combinations of BRAF/RAS with TERT, PIK3CA, or TP53, inform decisions to extend surgical interventions to encompass lobectomy and ipsilateral central lymph node dissection in differentiated thyroid cancer, assessing recurrence risk and tailoring surgical strategies [20]. Furthermore, RET fusions testing aids in the therapeutic application of vandetanib or cabozantinib [21], NTRK testing supports the utilization of larotrectinib and entrectinib [22], and BRAF^V600E^ testing directs therapy with dabrafenib and trametinib [23]. The application of NGS to detect these genetic alterations is pivotal in the diagnosis and prognosis of thyroid carcinoma, facilitating the development of personalized treatment strategies.

## 2. Materials and Methods

### 2.1. Patient Characteristics

This investigation entailed a retrospective analytical review of TNs patients who underwent primary surgical interventions at the Sun Yat-sen Memorial Hospital, Sun Yat-sen University (SYSMH), spanning the period from 1 January 2021, to 31 December 2023. The study included patients who met the following criteria: (1) underwent thyroid surgery and cervical lymph node dissection, (2) underwent routine ultrasound examination in our hospital before surgery, (3) had a thyroid cancer biopsy with fine needle aspiration before surgery, and (4) underwent NGS genetic testing of samples from preoperative fine-needle aspiration biopsy. Patients with the following conditions should be excluded: (1) cases in which fine-needle aspiration biopsy failed; (2) cases in which the sample size of a fine needle aspiration biopsy is not sufficient for NGS; (3) patients who have not undergone lymph node dissection during surgery; (4) patients with lymph node tuberculosis, primary lymph node disease, or metastasis of other tumors to lymph nodes; and (5) patients with a history of surgery or radiotherapy for other neck tumors. Concurrently, a comprehensive compilation of patient demographic information, ultrasonic characteristics, and NGS results was meticulously assembled. All patients only have one sample. The ethical considerations of this study were rigorously adhered to, with the research protocol obtaining prior approval from the Ethics Committee of the Sun Yat-sen Memorial Hospital, Sun Yat-sen University (Ethics number SYSKY-2024-658-01). Enrolment of participants was predicated upon the receipt of their written informed consent, thereby upholding the principles of autonomy and ethical conduct in research.

The analysis was extended to encompass a spectrum of TN subtypes, including BT, LRN, PTC, FTC, PDTC&ATC, and MTC. The clinical characteristics and NGS reports for each patient cohort were systematically scrutinized to elucidate the molecular and phenotypic correlates within these distinct thyroid tumorigenesis categories.

### 2.2. Tumor Selection and Processing

Ultrasound examinations were conducted by two radiologists, each possessing over five years of expertise in the US and a minimum of three years in FNAC. The US examinations were performed utilizing high-frequency (6–10 MHz) linear array probes in conjunction with an ACUSON Sequoia scanner (Siemens Medical Solutions, Ann Arbor, MI, USA). The ultrasonographic assessments were conducted in accordance with the 2017 ACR Thyroid Imaging Reporting and Data System (TI-RADS), evaluating parameters such as composition, echogenicity, shape, margin, and echogenic foci. The ACR classification of TNs is illustrated in Supplementary Table S1.

FNAC procedures were executed utilizing a 22-to-23-gauge needle procured from Hakko Corporation Ltd., Chikuma-Shi, Nagano, Japan, with a minimum of three aspirations conducted per thyroid lesion. The aspirated material was immediately injected into a FNAC preservation medium and processed via a liquid-based preparation (LBP) cell sedimentation automated slide preparation system. Each slide was meticulously prepared with 200 μL of the cell suspension and subsequently stained with H&E for histopathological examination. The Bethesda classification of TNs is shown in Supplementary Table S2.

Any residual biopsy samples were rapidly preserved on dry ice at temperatures below −60 °C and then expeditiously transported to the laboratory for DNA extraction. Extraction of DNA from cytology specimens was facilitated using the QIAamp DNA FFPE Tissue Kit (Qiagen, Valencia, CA, USA). The integrity and purity of the extracted DNA samples were rigorously assessed employing a Qubit fluorometer (Thermo Fisher Scientific Inc., Waltham, MA, USA). Thereafter, the DNA samples underwent sequencing utilizing a Nextseq500 sequencer (Illumina, San Diego, CA, USA), enabling the identification of genetic markers correlated with thyroid carcinoma.

### 2.3. Next-Generation Sequencing

For the genomic analysis of thyroid carcinoma, DNA libraries were meticulously prepared and subsequently sequenced employing a Nextseq500 sequencer (Illumina), utilizing a paired-end reads protocol. This high-throughput sequencing approach facilitated a comprehensive investigation of genes implicated in thyroid carcinoma. The analytical strategy encompassed the deployment of three distinct panels: an 18-gene panel, an 88-gene panel, and a broad-spectrum panel of thyroid carcinoma-related genes, all of which have been previously validated for their efficacy in discerning genomic alterations. These panels were strategically designed to encompass a selection of DNA mutations such as ALK, BRAF, HRAS, KRAS, NRAS, PIK3CA, PTEN, RET, and TERT promoter genes (Supplementary Table S3). The numbers of each tumor type in each panel are shown in Supplementary Table S4. This multi-tiered approach allowed for an exhaustive examination of the genetic landscape associated with thyroid carcinoma, providing a robust foundation for the identification of molecular signatures and potential therapeutic targets.

### 2.4. Data Analysis

Data were summarized as frequencies (percentages) for categorical variables and medians (interquartile ranges) for continuous variables, as appropriate for non-normally distributed data. Initial exploratory analysis included testing for normality using the Shapiro–Wilk test and visual inspection of Q–Q plots. Group comparisons were performed using non-parametric tests (Mann–Whitney U for two groups; Kruskal–Wallis with Dunn’s post hoc correction for ≥3 groups). Associations were assessed via Fisher’s exact test or chi-square test with Yates’ continuity correction (applied where expected cell counts were <5). To mitigate Type I error inflation from multiple comparisons, *p*-values were adjusted using the Benjamini–Hochberg false discovery rate (FDR). Only FDR-adjusted *p*-values < 0.05 were deemed significant.

Key clinical outcomes (e.g., nodal metastasis, tumor size > 4 cm) were further analyzed using multivariable logistic regression to adjust for confounders (age, sex, multifocality). Effect sizes were reported as odds ratios (ORs) with 95% confidence intervals (CIs). Model assumptions (e.g., absence of multicollinearity via variance inflation factors < 5) were rigorously verified.

All analyses were conducted in R v4.2.3 (stats, rstatix, and fmsb packages). Genetic alteration profiles were visualized using cBioPortal’s MutationMapper and customized via ggplot2 (R). Transparency was ensured by archiving analysis scripts on GitHub (Desktop 3.5.0).

## 3. Results

### 3.1. Patient Characteristics

**Figure 1:**
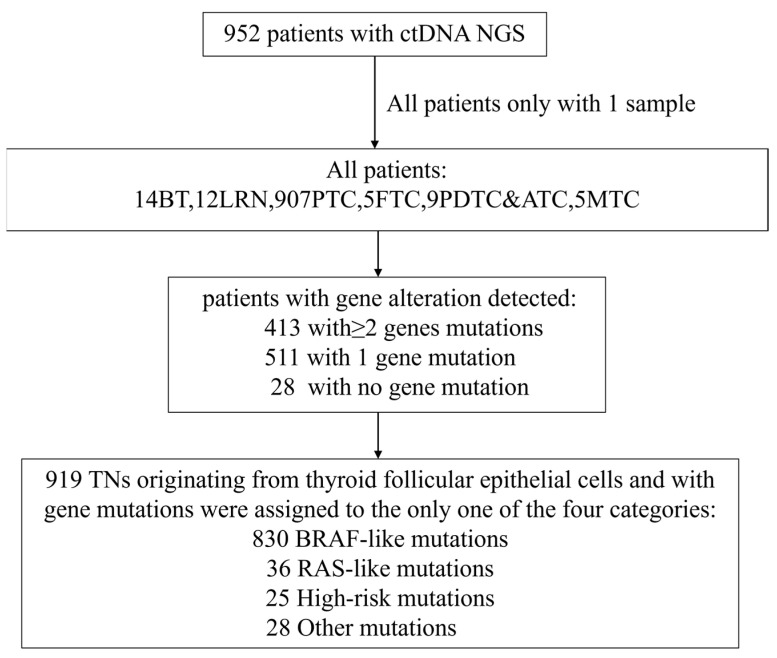
Study flow chart. In total, 952 patients were enrolled in this trial. Among them, 14 patients were BT, 12 were LRN, 907 were PTC, 5 were FTC, 9 were PDTC&ATC and 5 were MTC. A total of 413 patients were detected with more than one gene mutation, 511 were detected with one gene mutation while 28 were detected with no gene mutation. Patients were stratified into only one of the four exclusive categories: BRAF-like (*n* = 830), RAS-like (*n* = 36), high-risk (*n* = 25) and other mutations (*n* = 28), followed by a comparative analysis of clinical characteristics across these categories.

**Figure 2:**
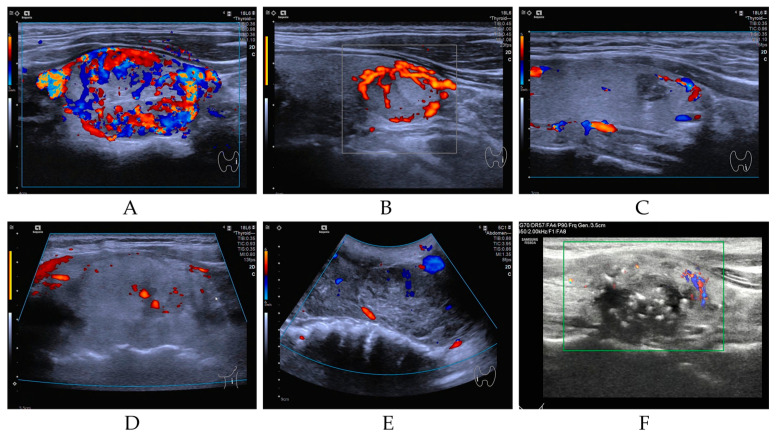
Representative ultrasonographic features of BT (**A**), LRN (**B**), PTC (**C**), FTC (**D**), PDTC&ATC (**E**), and MTC (**F**).

Our study cohort encompassed a total of 952 individuals diagnosed with a spectrum of thyroid neoplasms, categorized as follows: BT (*n* = 14, 1.47%), LRN (*n* = 12, 1.26%), PTC (*n* = 907, 95.27%), FTC (*n* = 5, 0.53%), PDTC&ATC (*n* = 9, 0.95%), and MTC (*n* = 5, 0.53%). As illustrated in Figure 1 (Study flow chart), all patients underwent DNA-NGS, with 919 follicular-derived tumors classified into four molecular categories: BRAF-like (*n* = 830, 90.3%), RAS-like (*n* = 36, 3.9%), high-risk (*n* = 25, 2.7%), and other mutations (*n* = 28, 3.1%). All pathological diagnoses were authenticated at the Sun Yat-sen Memorial Hospital, ensuring the accuracy of our study’s foundational data. The NGS database incorporated data extracted from 952 distinct samples, each aligning with an individual patient. Figure 2 presents representative ultrasonographic features of BT, LRN, PTC, FTC, PDTC&ATC, and medullary thyroid carcinoma (MTC).

Demographic characteristics of the cohort are delineated in Table 1. The median age within the cohort was 41 years, spanning a range from 8 to 76 years, with 77.63% of participants identified as female. No significant disparities in sex distribution were observed across the neoplasm subtypes. Comparative analysis of age among the neoplasm categories revealed no significant differences, with the exception of the FTC and PDTC&ATC groups, wherein patients exhibited a higher mean age compared to other groups (*p* < 0.001).

The mean maximum diameter of PTC tumors was recorded at 11.06 mm, with a variance from 1 to 70 mm. This measurement was markedly smaller in comparison to the BT group, which exhibited a mean diameter of 32.00 mm (*p* < 0.001), the LRN group with a mean of 23.43 mm (*p* < 0.001), the MTC group with 20.00 mm (*p* = 0.135), and the PDTC&ATC group with 54.00 mm (*p* < 0.001). No statistically significant variation in tumor size was observed in relation to the FTC group, which demonstrated a mean diameter of 13.50 mm (*p* = 0.495).

Lymph node metastasis was not detected in the BT group. Conversely, the rate of lymph node metastasis within the PTC group was 58.54%, which was substantially lower than the rates observed in the PDTC&ATC (66.67%) group. These findings underscore the heterogeneity in the biological behavior of different thyroid neoplasm subtypes and provide insights into the prognostic implications of these variations.

### 3.2. Characterization of Genomic Alterations in Thyroid Neoplasms

In a comprehensive genomic analysis of 952 FNAC samples derived from a diverse spectrum of thyroid neoplasms, we employed targeted NGS to interrogate the mutational landscape. Specifically, 328 samples were subjected to a panel encompassing 18 thyroid carcinoma-related genes, while an extended panel of 88 thyroid carcinoma-related genes was utilized for an additional 607 samples. A select cohort of 17 samples underwent comprehensive analysis with a panel encompassing broad-spectrum panel related genes (refer to Supplementary Table S3 for details). Our analysis revealed the presence of gene mutations in 924 patients, with a subset of 413 patients exhibiting multiple gene mutations. Of particular note, 28 patients, including those with BT (*n* = 4), LRN (*n* = 1), PTC (*n* = 21), FTC (*n* = 1), and PDTC&ATC (*n* = 1), displayed no detectable mutations.

**Figure 3:**
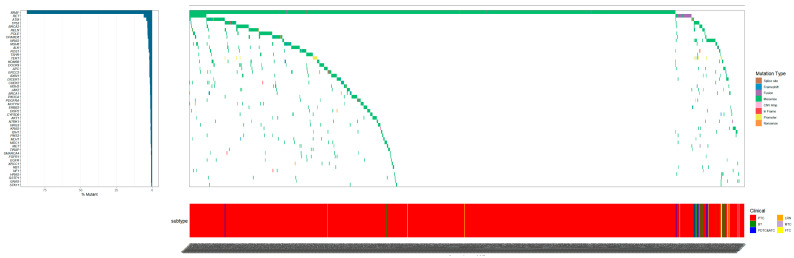
Oncoprints of the 952 thyroid neoplasms patients in our study. The top 50 mutated genes are listed on the left of the mutation spectrum. The color details for genetic alterations and histology are shown on the right outer part.

The mutational analysis identified a total of 924 distinct mutations, with BRAF^V600E^ emerging as the most prevalent, affecting 804 individuals (84.45%). Other frequently mutated genes included RET (*n* = 61, 6.41%), BRCA1/2 (*n* = 42, 4.41%), RAS (*n* = 42, 4.41%), ATM (*n* = 38, 3.99%). A detailed enumeration of the top 20 genomic alterations across the thyroid neoplasm subtypes is presented in Table 2, with the top 50 alterations graphically represented in Figure 3.

The RET proto-oncogene encodes a transmembrane receptor tyrosine kinase and the alteration results in ligand-independent signaling and oncogenesis. RET mutations are commonly found in MTC. Despite only five cases, the incidence of RET mutations in MTC remains at 20%, which is higher than in other types of thyroid cancer (Table 3). RET mutations are linked to more aggressive medullary thyroid cancer (MTC), leading to metastasis in a majority of patients [24]. MTC patients showed an earlier age of onset (37 years vs. 42 years), larger tumor diameter (20.00 mm vs. 11.06 mm), and more frequent lymph node metastasis (60.00% vs. 58.54%) compared to PTC patients (Table 1). Moreover, RET fusions occur in less than 10% of non-medullary thyroid carcinoma cases [25,26]. The RET mutation rate was 11.11% (1 case out of 9) in ATC cases, 6.51% (59 cases out of 907) in PTC cases, and 0% in BT, LRN, or FTC (Table 3).

### 3.3. Genomic Alterations of Four Mutation Types in Thyroid Neoplasms

The 5th edition of the WHO classification of those thyroid tumors originating from thyroid follicular epithelial cells, integrating morphological characteristics, mutations, and transcriptomic features, distinguishes RAS-like and BRAF-like tumors. Molecular abnormalities in RAS-like tumors include mutations in BRAF K601E, DICER1, EZH1, EIF1AX, and fusions in PPARG, THADA, whereas BRAF-like molecular abnormalities encompass fusions in ALK, BRAF, RET, NTRK1/3, and MET. BRAF-like tumors are generally associated with poorer prognosis, linked to higher recurrence rates and invasive behavior [27,28]. The prognosis of RAS-like tumors may differ from that of BRAF-like tumors, and understanding these differences aids in more accurate prediction of disease progression in patients. Distinguishing between RAS-like and BRAF-like tumors is crucial for formulating effective treatment plans, assessing disease progression, and improving patient outcomes. With the advancement of molecular diagnostic technologies, this distinction is becoming increasingly precise, facilitating more targeted medical management. The 2015 ATA guidelines indicate that BRAF mutations, in combination with other gene mutations such as TERT, PIK3CA, and TP53, suggest a higher risk of malignancy in thyroid cancer, thus categorizing them as high-risk mutations [29]. Mutations not fitting these classifications are labeled as “other mutations”. Table 4 summarizes the gene mutations detected by NGS across the four categories of thyroid neoplasms (TNs).

Patients were exclusively stratified into four mutation categories (high-risk, BRAF-like, RAS-like, and other) for comparative analysis. High-risk mutations showed strong male predominance (48.00% vs. 21.45% BRAF-like, *p* = 0.008; odds ratio, OR = 3.25) and older age distribution (52.00% ≥ 55 y vs. 12.65% BRAF-like, *p* < 0.001; OR = 7.50), with tumors more frequently >4 cm (19.05% vs. 1.33% BRAF-like, *p* < 0.001; OR = 17.45) and multifocal (96.00% vs. 43.26% BRAF-like, *p* < 0.001; OR = 30.67). Notably, extra-thyroid extension was significantly higher in high-risk versus BRAF-like groups (12.00% vs. 3.13%, *p* = 0.022; OR = 0.23), while nodal metastases showed no intergroup differences (*p* = 0.940). Tumor size distributions differed markedly, with high-risk cases exhibiting smaller (≤1 cm: 71.43% vs. 38.48% BRAF-like, *p* = 0.005) yet more invasive characteristics (Table 5).

### 3.4. Association of BRAF/RAS and TERT Co-Mutations with Tumor Size and Lymph Node Metastasis in Thyroid Carcinoma

**Figure 4:**
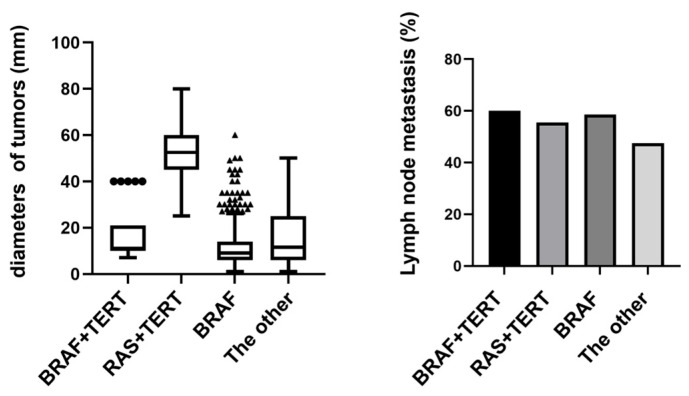
Tumor size and lymph node metastasis in BRAF+TERT vs. RAS+TERT mutations. Tumors with BRAF+TERT co-mutations showed higher lymph node metastasis rates, while RAS+TERT co-mutations were associated with larger tumor size, suggesting distinct roles of these mutations in tumor aggressiveness.

Our stratified analysis of high-risk mutation combinations revealed significant differences in tumor characteristics across molecular subgroups (Figure 4). Tumors harboring BRAF+TERT co-mutations demonstrated particularly aggressive features, with 60% lymph node metastasis (6/10 cases) and a mean tumor diameter of 16.8 mm. Notably, the RAS+TERT group, while showing comparable metastasis rates (55.6%, 5/9 cases), exhibited substantially larger tumor diameters (mean 56 mm), suggesting potential mechanistic differences in how these mutations influence tumor growth patterns. Mutation-negative tumors presented the most favorable profile with the lowest metastasis rate (42.9%, 12/28 cases), though their intermediate tumor size (mean 18.6 mm) indicates additional factors may regulate growth independent of these driver mutations. These findings collectively underscore the clinical importance of molecular stratification, particularly identifying TERT co-mutations as potential markers of aggressive disease.

### 3.5. Assessment of Tumor Mutation Burden in Thyroid Neoplasms

**Figure 5:**
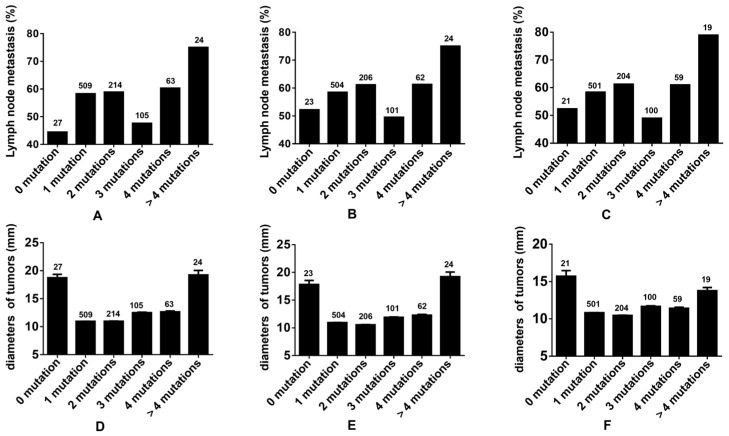
Evaluating tumor mutation burden in thyroid tumors. (**A**,**D**): The incidence rates of lymph node metastasis and the diameters of tumors associated with various number gene mutations in all thyroid tumors. (**B**,**E**): The incidence rates of lymph node metastasis and the diameters of tumors associated with various number gene mutations in all thyroid malignant tumors. (**C**,**F**): The incidence rates of lymph node metastasis and the diameters of tumors associated with various number gene mutations in all PTCs.

The rate of lymph node metastasis within the cohort devoid of gene mutations was 57.14% across all thyroid tumors. This rate escalated with an increment in the multiplicity of gene mutations, with the exception of the group presenting three gene mutations. Analogous trends were identified in both malignant thyroid tumors and PTCs. Tumor diameters were observed to augment in tandem with the ascending number of gene mutations within the mutated groups (Figure 5). Notably, the tumors in the gene mutation-negative group did not exhibit the smallest dimensions. Corresponding tendencies were also observed in cases of malignant thyroid tumors and PTCs.

## 4. Discussion

The rising global burden of thyroid cancer has spurred significant interest in molecular diagnostics, particularly for risk stratification and personalized management [30,31]. While recent NGS-based studies [32,33,34] have demonstrated clinical utility in specific scenarios—such as indeterminate cytology or active surveillance—our work advances the field by establishing a comprehensive molecular framework tailored to the unique epidemiological and clinical landscape of Chinese thyroid cancer patients. Unlike previous studies that focused primarily on diagnostic performance, our analysis of 952 FNA samples not only confirms the high prevalence of PTC (95.27%) in China but also reveals distinct molecular patterns that directly inform therapeutic decisions. For instance, our identification of RET/NTRK fusions in 10.4% of PTC cases and BRAF/TERT co-mutations in high-risk subgroups provides actionable therapeutic targets, bridging the gap between molecular profiling and clinical management in ways that previous studies did not fully explore.

Furthermore, our study significantly enhances preoperative risk stratification by correlating mutational profiles with aggressive clinical features. Whereas Ren et al. reported BRAF mutations in 81.9% of Chinese PTCs without connecting them to specific management pathways, our multivariate analyses demonstrate that BRAF-like mutations are strongly associated with lymph node metastasis (OR = 3.25, *p* = 0.008) and tumor size > 4 cm (OR = 17.45, *p* < 0.001). Importantly, our improved panel design, which includes CNAs and novel fusion detection, outperformed prior studies in diagnostic accuracy for indeterminate nodules (79.2% sensitivity vs. Ren et al.’s 44.4% NPV). These findings address a critical unmet need in thyroid nodule management—moving beyond mere diagnosis to predict disease behavior and optimize treatment selection. Unlike Potonnier’s limited NPV (87%) or Ramone’s focus on low-risk microcarcinomas, our risk-stratified approach enables clinicians to confidently triage patients toward surveillance, targeted therapy, or surgery.

Ultimately, our work transcends the “replication” critique by demonstrating how DNA-based NGS from FNA samples can revolutionize thyroid cancer care in real-world settings. By validating clinically actionable mutation categories and refining multivariate risk models, we provide a template for integrating molecular insights into routine practice—precisely the “novel clinical management insights” the reviewer demanded. Future studies should build on this framework to further validate its impact on reducing unnecessary surgeries while ensuring precise targeting of advanced therapies, particularly in high-risk populations that traditional algorithms might miss. This represents a meaningful step toward personalized medicine in thyroid cancer, transforming molecular data into tangible improvements in patient outcomes.

In the realm of thyroid cancer pathogenesis and biological research, considerable progress has been achieved in recent years. A plethora of studies has underscored the significance of BRAF^V600E^ and RAS mutations in the initiation phase of thyroid cancer [4]. The perpetuation of BRAF^V600E^ and RAS mutations, coupled with the emergence of additional mutations in PDTC and ATC, is instrumental in the transition of thyroid cancer from a well-differentiated to a poorly differentiated, and ultimately, an undifferentiated state [35]. Furthermore, alterations within the TERT promoter, TP53, PIK3CA, MMR, and histone methyltransferase (HMT) pathways are identified as playing a critical role in the differentiation and progression of tumors [36].

In this study, the BRAF^V600E^ mutation was the most prevalent, found in 84.45% of cases. In patients with PTC, BRAF^V600E^ was detected in 88.53%, respectively. The high BRAF^V600E^ mutation rate is attributed to the predominance of PTC in our cohort, with PTMC accounting for approximately 90% of these. In Asian populations, BRAF^V600E^-mutated thyroid nodules carry a 99.8% malignancy risk, and the mutation has a very low false-positive rate. Thus, BRAF^V600E^ detection in FNA cytology specimens strongly indicates a malignant nodule. If BRAF^V600E^ mutation is identified preoperatively, the procedure should include lymph node dissection or total thyroidectomy due to the mutation’s association with tumor recurrence. Recurrent thyroid cancers with central lymph node metastasis have BRAF^V600E^ mutation rates ranging from 78% to 95%, supporting the recommendation for prophylactic central lymph node dissection in BRAF^V600E^-mutated patients.

The rates of detected RAS by FNAC samples were similar in our study compared to historic cohorts [14,37]. Although some data suggest that RAS gene mutations may increase the risk of malignancy, most RAS-positive thyroid cancers exhibit a low-risk phenotype. Thyroid nodules that are cytologically benign but RAS mutation-positive can remain inactive for many years [38,39]. If the RAS mutation is the sole mutation detected, the target thyroid nodule is likely of low malignancy (benign nodule, NIFTP, or low-risk thyroid cancer), potentially becoming a candidate for active surveillance, and if surgery is necessary, a lobectomy may suffice. However, if RAS mutations co-occur with other gene mutations, particularly in the TERT promoter, EIF1AX, or TP53, the risk of highly aggressive thyroid cancer significantly increases [40]. In summary, if RAS gene mutations are detected in FNA samples, especially as the only mutation, the thyroid cancer or nodule is likely of relatively low malignancy and good prognosis, and non-surgical treatment and conservative observation can be considered. When other types of mutations are combined, special attention is warranted, and more aggressive treatment modalities should be considered.

Among the mutations identified, thyroid neoplasms were classified into BRAF-like, RAS-like genotypes, high-risk mutations, and other mutations that do not fit into the above categories. The TCGA study indicates that 90% of PTC are driven by the activation of the MAPK pathway, which occurs through mutually exclusive mutations in BRAF or RAS oncogenes [35,41]. In our study, 25 cases were found to have co-occurring BRAF^V600E^ and NRAS mutations [42], and additionally, 10 cases presented compound mutations involving BRAF^V600E^ with TERT promoter and TP53 genes [43], suggesting that both can occur rarely. A higher incidence of nodal metastases was observed in TNs with BRAF-like mutations relative to RAS-like mutations. The rates of lymph node metastasis were comparable between the BRAF-like and high-risk groups.

When BRAF-like alterations are present, the likelihood of cancer is extremely high, with a higher risk of metastasis and early recurrence, typically seen in PTC. In contrast, RAS-like alterations suggest a lower likelihood of cancer and a lower risk of recurrence, often associated with follicular-structured carcinomas or noninvasive follicular thyroid neoplasms with NIFTP, and benign adenomas. TERT promoter mutations are associated with tumor progression, invasive behavior, and poor clinical outcomes. The co-occurrence of TERT promoter mutations and BRAF-like mutations indicates an extremely high risk of thyroid cancer and is associated with increased extrathyroidal spread and lymph node metastasis risk. This combined occurrence is more frequently observed in poorly differentiated and anaplastic thyroid carcinomas [44]. Co-detection of BRAF, RAS, RET, TERT promoter, and TP53 in preoperative fine-needle aspiration cytology specimens aids in assessing and predicting the biological behavior of thyroid cancer, effectively guiding tumor recurrence risk stratification, screening for multiple endocrine neoplasia (MEN)-related thyroid carcinomas, and providing a basis for systemic targeted therapy decisions.

Furthermore, we found no direct correlation between the number of genetic mutations in thyroid cancer and tumor size, despite a numerical trend, and there was no statistically significant difference. The size of thyroid cancer tumors may be influenced by a variety of factors, including but not limited to genetic mutations, the growth rate of the tumor, and the overall health status of the patient. For patients with thyroid cancer, understanding the genetic mutation profile is instrumental in formulating more precise treatment plans, but the size of the tumor still needs to be assessed through imaging and other clinical examinations [45].

Our rationale for employing a broad gene panel stems from emerging evidence that thyroid cancer pathogenesis involves complex genomic landscapes beyond canonical drivers like BRAF and RAS. While BRCA mutations are primarily associated with breast/ovarian cancer, recent studies have identified unexpected somatic BRCA alterations in 3–5% of thyroid carcinomas, particularly in aggressive variants [46,47]. Though not yet incorporated into routine diagnostic pathways, these findings suggest potential shared oncogenic mechanisms that warrant exploration.

Despite the valuable insights gained, our study acknowledges several limitations. The most prominent of these is the overrepresentation of PTC in our cohort, which skews our findings and may prevent us from fully capturing the biological characteristics of the entire spectrum of thyroid tumors. The predominance of PTC cases means that our results may not be generalizable to other thyroid malignancies with distinct molecular profiles and clinical behaviors. A related methodological constraint is the variability in NGS panel coverage across cases. Due to the retrospective nature of this study and evolving clinical protocols, 328 cases were analyzed using an 18-gene panel, 607 with an 88-gene panel, and 17 with a broader pan-cancer panel. While this allowed us to capture major driver mutations (e.g., BRAF, RAS, fusions) consistently across all panels, differences in gene coverage could have led to under-detection of mutations, particularly in rarer non-PTC subtypes. Although subgroup analyses showed no significant divergence in mutation rates between panel types, future prospective studies should adopt standardized, expanded panels to ensure uniform sensitivity across all thyroid tumor types. Additionally, there were inherent shortcomings in data collection and analysis. Retrospective designs may introduce biases due to inconsistent medical records or missing data. While we performed rigorous quality control, some confounding factors (e.g., treatment heterogeneity, sample preservation) could not be fully adjusted for. These limitations highlight the need for multicenter validation with harmonized sequencing protocols to refine our molecular stratification framework.

To mitigate these issues in future studies, it would be beneficial to include a more diverse and balanced representation of thyroid tumor types. Utilizing more comprehensive NGS panels that cover a broader range of genes associated with various thyroid tumors could help in identifying a more complete set of genetic mutations. Moreover, improving data collection protocols and employing more rigorous analytical techniques will be crucial in enhancing the quality of the data and the validity of the conclusions drawn.

In summary, while our study provides valuable insights into the genetic mutations associated with thyroid tumors, particularly PTC, the noted limitations highlight the need for caution in interpreting our results. Future research should aim to address these shortcomings by adopting more inclusive study designs, employing advanced genetic analysis tools, and refining data analysis methods to provide a more comprehensive and accurate understanding of the genetic underpinnings of thyroid tumors.

## 5. Conclusions

In conclusion, our study reaffirms the integral role of molecular profiling in thyroid cancer before operation and highlights the need for further validation and exploration of the genetic underpinnings of this disease. By continuing to unravel the complex genetic architecture of thyroid neoplasms, we move closer to realizing personalized medicine for patients with thyroid cancer.

## Figures and Tables

**Table 1:** Clinical characteristics of the cohort.

	Total	BT	LRN	PTC	FTC	PDTC&ATC	MTC
Number of patients	952	14 (1.47%)	12 (1.26%)	907 (95.27%)	5 (0.53%)	9 (0.95%)	5 (0.53%)
Gender, male (%)	213 (22.37%)	6 (42.86%)	3 (25.00%)	196 (21.61%)	3 (60.00%)	6 (66.67%)	0
Age, mean (years, range)	41 (8.76)	41 (12.70)	43 (18.66)	42 (8.76)	58 (44.69)	61 (50.76)	37 (13.49)
Tumor size, mean (mm, range)	11.12 (1.70)	32.00 (24.40)	23.42 (8.50)	11.06 (1.70)	13.50 (1.25)	54.00 (21.80)	20.00 (5.40)
Lymph node metastasis, *n* (%)	544 (57.14%)	0	2 (16.67%)	531 (58.54%)	2 (40.00%)	6 (66.67%)	3 (60.00%)

BT—benign tumors; LRN—low risk neoplasms; PTC—papillary thyroid carcinoma; FTC—follicular thyroid carcinoma; PDTC&ATC—poorly differentiated thyroid carcinoma and anaplastic thyroid carcinoma; MTC—medullary thyroid carcinoma.

**Table 2:** Top 20 Genomic alterations detected in different types of thyroid neoplasms.

Genes	Total	BT	LRN	PTC	FTC	PDTC&ATC	MTC
N	952	14	12	907	5	9	5
No mutations	28 (2.94%)	4 (28.57%)	1 (8.33%)	21 (2.32%)	1 (20.00%)	1 (11.11%)	0
BRAF^V600E^	804 (84.45%)			803 (88.53%)		1 (11.11%)	
RET	61 (6.41%)			59 (6.51%)		1 (11.11%)	1 (20.00%)
BRCA1/2	42 (4.41%)			41 (4.52%)		1 (11.11%)	
N/H/K-RAS	42 (4.41%)	4 (28.57%)	4 (33.33%)	26 (2.87%)	2 (40.00%)	6 (66.67%)	
ATM	38 (3.99%)		1 (8.33%)	37 (4.08%)			
RELN	30 (3.15%)	2 (14.29%)		26 (2.87%)		2 (22.22%)	
POLE	30 (3.15%)			29 (3.20%)	1 (20.00%)		
TP53	29 (3.05%)			23 (2.54%)		6 (66.67%)	
CPAMD8	28 (2.94%)			28 (3.09%)			
MSH6	25 (2.63%)	1 (7.14%)		22 (2.43%)		2 (22.22%)	
ALK	24 (2.52%)		1 (8.33%)	23 (2.54%)			
TERT	21 (2.21%)			13 (1.43%)	2 (40.00%)	6 (66.67%)	
ROS1	21 (2.21%)	1 (7.14%)		20 (2.21%)			
TSHR	18 (1.89%)			18 (1.98%)			
KDM6B	18 (1.89%)	2 (14.29%)		16 (1.76%)			
DOCK9	18 (1.89%)			18 (1.98%)			
APC	17 (1.79%)	2 (14.29%)		14 (1.54%)		1 (11.11%)	
AXIN1	17 (1.79%)	1 (7.14%)		16 (1.76%)			
ERCC2	16 (1.68%)			16 (1.76%)			
CHEK1/2	16 (1.68%)	1 (7.14%)		15 (1.65%)			

BT—benign tumors; LRN—low risk neoplasms; PTC—papillary thyroid carcinoma; FTC—follicular thyroid carcinoma; PDTC&ATC—poorly differentiated thyroid carcinoma and anaplastic thyroid carcinoma; MTC—medullary thyroid carcinoma.

**Table 3:** RET mutation profiling by NGS in thyroid FNA samples across tumor types.

*RET* Alteration	BT	LRN	PTC	FTC	PDTC&ATC	MTC
N	14	12	907	5	9	5
Fusion			28 (3.09%)			
A1051V			1 (0.11%)			
A144H			1 (0.11%)			
A475T			1 (0.11%)			
A540G					1 (11.11%)	
A587T			1 (0.11%)			
A1042A			1 (0.11%)			
C630R						1 (20.00%)
G136L			1 (0.11%)			
G568S			1 (0.22%)			
G691S			1 (0.11%)			
G823V			1 (0.11%)			
G830A			3 (0.33%)			
L481V			4 (0.44%)			
P384A			1 (0.11%)			
R348Q			1 (0.11%)			
R694Q			1 (0.11%)			
T1284M			1 (0.11%)			
T606H			1 (0.11%)			
V292M			1 (0.11%)			
V351G			1 (0.11%)			
G568S			1 (0.11%)			
P854S			1 (0.11%)			
A600T			1 (0.11%)			
G136L			1 (0.11%)			
V388I			1 (0.11%)			
A982H			1 (0.11%)			
V804M			1 (0.11%)			
Total (%)			59 (6.51%)		1 (11.11%)	1 (20.00%)

BT—benign tumors; LRN—low risk neoplasms; PTC—papillary thyroid carcinoma; FTC—follicular thyroid carcinoma; PDTC&ATC—poorly differentiated thyroid carcinoma and anaplastic thyroid carcinoma; MTC—medullary thyroid carcinoma.

**Table 4:** Genomic alterations of four mutation types in FNAC samples from various thyroid neoplasms.

Mutations Number (%)		BT	LRN	PTC	FTC	PDTC&ATC	MTC
N		14	12	907	5	9	5
BRAF-like mutations	BRAF^V600E^			803 (88.53%)		1 (11.11%)	
	BARF fusions			4 (0.44%)			
	RET fusions			28 (3.09%)			
	RET (point mutation, indel, other)			31 (3.42%)		1 (11.11%)	1 (20.00%)
	NTRK1/3 fusions	1 (7.14%)		4 (0.44%)			
	ALK		1 (8.33%)	23 (2.54%)			
	MET			7 (0.77%)		1 (11.11%)	
RAS-like mutations	RAS (N/H/K-Ras)	4 (28.57%)	4 (33.33%)	26 (2.87%)	2 (40.00%)	6 (66.67%)	
	BRAF K601E			2 (0.22%)			
	EIF1AX	1 (7.14%)				3 (33.33%)	
	EZH1			3 (0.33%)		1 (11.11%)	
	DICER1	2 (14.29%)	1 (8.33%)	9 (0.99%)			1 (20.00%)
	PTEN			3 (0.33%)		2 (22.22%)	
	TSHR			18 (1.98%)			
	PPARGR fusions						
	THADA fusions						
high-risk mutations	TP53			23 (2.54%)		6 (66.67%)	
	TERT promoter			13 (1.43%)	2 (40.00%)	6 (66.67%)	
	PIK3CA			11 (1.21%)	2 (40.00%)	1 (11.11%)	
Other mutations	APC	2 (14.29%)		14 (1.54%)		1 (11.11%)	
	ATM		1 (8.33%)	37 (4.08%)			
	AXIN1	1 (7.14%)		17 (1.87%)			
	AKT			12 (1.32%)			
	BRCA1/2			41 (4.52%)		1 (11.11%)	
	CHEK1/2	1 (7.14%)		15 (1.65%)			
	CPAMD8			28 (3.09%)			
	DOCK9			18 (1.98%)			
	ERBB2		1 (8.33%)	11 (1.21%)			
	ERCC2			16 (1.76%)			
	MLH1			8 (0.88%)	1 (20.00%)		
	MSH2/6	1 (7.14%)		33 (3.64%)		3 (33.33%)	
	POLE			29 (3.20%)	1 (20.00%)		
	RELN	2 (14.29%)		26 (2.87%)		2 (22.22%)	
	ROS1	1 (7.14%)		20 (2.21%)			
No mutations		4 (28.57%)	1 (8.33%)	21 (2.32%)	1 (20.00%)	1 (11.11%)	

BT—benign tumors; LRN—low risk neoplasms; PTC—papillary thyroid carcinoma; FTC—follicular thyroid carcinoma; PDTC&ATC—poorly differentiated thyroid carcinoma and anaplastic thyroid carcinoma; MTC—medullary thyroid carcinoma.

**Table 5:** Association of BRAF-like mutations, RAS-like mutations, high-risk mutations and other mutations.

Characteristic	BRAF-like Mutations	RAS-like Mutations	High-Risk Mutations	Other Mutations	BRAF-like vs. High-Risk	
	N = 830	N = 36	N = 25	N = 28	OR [95% CI]	*p*
Tumor Type						
BT	0	5 (13.89%)	0	5 (17.86%)		
LRN	0	8 (22.22%)	0	3 (10.71%)		
PTC	829 (99.88%)	22 (61.11%)	16 (64.00%)	19 (67.86%)		
FTC	0	0	3 (12.00%)	1 (3.57%)		
PDTC&ATC	1 (0.12%)	1 (2.78%)	6 (24.00%)	0		
Sex, *n* (%)						
Female	652 (78.55%)	27 (75.00%)	13 (52.00%)	22 (78.57%)	3.25 [1.42–7.45]	0.008
Male	178 (21.45%)	9 (25.00%)	12 (48.00%)	6 (21.43%)		
Years (range)	(8.76)	(13.70)	(14.76)	(21.63)		
≥55 y, *n* (%)						
No	725 (87.35%)	32 (88.89%)	12 (48.00%)	2 (89.29%)	7.50 [3.20–17.60]	0.000
Yes	105 (12.65%)	4 (11.11%)	13 (52.00%)	3 (10.71%)		
Tumor diameter (%)						
≤1 cm	319 (38.48%)	19 (54.29%)	15 (71.43%)	15 (53.57%)	0.24 [0.09–0.62]	0.005
>1 cm	510 (61.52%)	16 (45.71%)	6 (28.57%)	13 (46.43%)		
Tumor diameter (%)						
≤4 cm	818 (98.67%)	31 (88.57%)	17 (80.95%)	28 (100.00%)	17.45 [4.35–70.00]	0.000
>4 cm	11 (1.33%)	4 (11.43%)	4 (19.05%)	0 (0)		
Multifocality, *n* (%)						
No	471 (56.74%)	21 (58.33%)	1 (4.00%)	17 (60.71%)	30.67 [4.05–232.00]	0.000
Yes	359 (43.26%)	15 (41.67%)	24 (96.00%)	11 (39.28%)		
Nodal metastases, *n* (%)						
No	341 (41.23%)	25 (66.67%)	8 (42.11%)	12 (44.44%)	0.97 [0.41–2.30]	0.940
Yes	486 (58.77%)	12 (33.33%)	11 (57.89%)	15 (55.56%)		
Extra-thyroid extension *n* (%)						
No	804 (96.87%)	35 (97.22%)	22 (88.00%)	28 (100%)	0.23 [0.07–0.82]	0.022
Yes	26 (3.13%)	1 (2.78%)	3 (12.00%)	0		

## Data Availability

Data is contained within the article and Supplementary Materials.

## Supplementary Materials

The following supporting information can be downloaded at https://www.mdpi.com/article/10.3390/cancers17152429/s1, Supplementary Table S1: ACR classification of all thyroid nodules; Supplementary Table S2: Bethesda classification of all thyroid nodules; Supplementary Table S3: Patients were subjected to a panel encompassing 18, 88 or a broad-spectrum gene of thyroid carcinoma. Supplementary Table S4. Distribution of Thyroid Cancer Subtypes Across Different Gene Panels; Supplementary Table S5: BRAF^V600E^ Mutation Frequency, Lymph Node Metastasis, and Tumor Size by Mutation Burden.

## Abbreviations

The following abbreviations are used in this manuscript:

NGS	Next-Generation Sequencing
FNAC	Fine-Needle Aspiration Cytology
PTC	Papillary Thyroid Carcinoma
FTC	Follicular Thyroid Carcinoma
PDTC	Poorly Differentiated Thyroid Carcinoma
ATC	Anaplastic Thyroid Carcinoma
MTC	Medullary Thyroid Carcinoma
LRN	Low-Risk Neoplasms
BT	Benign Tumors
NIFTP	non-invasive follicular thyroid neoplasm with papillary-like nuclear features
