# Supplementary material for: Mutational Profiling Detection in FNAC Samples of Different Types of Thyroid Neoplasms Using Targeted NGS
Source: Cancers (Basel). 2025 Jul 23;17(15):2429. doi: 10.3390/cancers17152429 (PMC12346461; doi:10.3390/cancers17152429)
Supplement: Supplementary file 1 [file cancers-17-02429-s001.zip › cancers-3720217 Supplementary Table S1.pdf]

Supplementary Table S1. ACR classification of all thyroid nodules

ACR classification	BT	LRN	PTC	FTC	PDTC&ATC	MTC
**1**						
**2**	1（7.14%）					
**3**	4（28.57%）	5（41.67%）	15(1.65%)			1(20.00%)
**4**	7（50.00%）	3（25.00%）	232(25.58%)	2(40.00%)	2(22.22%)	1(20.00%
**5**	2（14.29%）	4(33.33%)	660(72.77%)	3(60.00%)	7(77.78%)	3(60.00%)

BT-benign tumors; LRN- low risk neoplasms; PTC - papillary thyroid carcinoma; FTC - follicular thyroid carcinoma; PDTC&ATC-poorly differentiated thyroid carcinoma and anaplastic thyroid carcinoma; MTC-medullary thyroid carcinoma.
