# Supplementary material for: Mutational Profiling Detection in FNAC Samples of Different Types of Thyroid Neoplasms Using Targeted NGS
Source: Cancers (Basel). 2025 Jul 23;17(15):2429. doi: 10.3390/cancers17152429 (PMC12346461; doi:10.3390/cancers17152429)
Supplement: Supplementary file 1 [file cancers-17-02429-s001.zip › cancers-3720217 Supplementary Table S2.pdf]

Supplementary Table S2. Bethesda classification of all thyroid nodules

Bethesda classification	BT	LRN	PTC	FTC	PDTC&ATC	MTC
I						
II	2（14.29%）	1(8.33%)				
III	5（35.71%）	5(41.67%)	16(1.76%)	1(20.00%		
IV	7（50.00%）	3(25%)		2(40.00%)		
V		1(8.33%)	24(2.65%)			
VI		2(16.67%)	867(95.59%)	2(40%)	9(100.00%）	5（100.00%）

BT-benign tumors; LRN- low risk neoplasms; PTC - papillary thyroid carcinoma; FTC - follicular thyroid carcinoma; PDTC&ATC-poorly differentiated thyroid carcinoma and anaplastic thyroid carcinoma; MTC-medullary thyroid carcinoma.
