# Supplementary material for: Mutational Profiling Detection in FNAC Samples of Different Types of Thyroid Neoplasms Using Targeted NGS
Source: Cancers (Basel). 2025 Jul 23;17(15):2429. doi: 10.3390/cancers17152429 (PMC12346461; doi:10.3390/cancers17152429)
Supplement: Supplementary file 1 [file cancers-17-02429-s001.zip › cancers-3720217 Supplementary Table S3.pdf]

Supplementary Table S3. Patients were subjected to a panel encompassing 18, 88 or a broad-spectrum genes of thyroid carcinoma.

Gene project	Number	List
**18 genes**	**328**	AKT1 、ALK 、BRAF 、CTNNB1 、EIF1AX 、ETV6 、 GNAS 、HRAS 、KRAS 、NRAS 、NTRK1 、 PIK3CA 、PPARG 、PTEN 、RET 、TERT 、TP53 、TSHR
**88 genes**	**607**	AAAS、BRAF、CTNNB1、ERCC1、HRAS、MET、NRAS、PTPN11 、SLC15A2 、TP53、ABCB1、CDH1、CYP1B1、ERCC2、IDH1、METTL14、NRG3、RAB42、SLC26A3、TSHR、AKT1、CDK5RAP2、DDX3X、ETV6、JAK2、MLH1、NTRK1、RANBP17 、SMAD4 、VHL、ALK、CDKN2A 、 DICER1 、 FAM81B 、 KDM6B 、MRPL50、OR52D1、RB1、SMARCA4、XRCC1、ALMS1、CDKN2C、DISP2、FAT4、KRAS、MSH2、OVCH1、RELN、SPTBN5、ZAN、APC、CHEK1、DOCK9、FBXW7、KRTAP10-5、MSH6、PIK3CA、RET、STK11、ZNF292、ASPM、CHEK2、EGFR、GABRA2、 LPA、 MYH7、PPARG、SEMA6A、TDG、ZNF878、ATM、CHGA、EIF1AX、GNAS、MDC1、NF1、PPM1D、SF3B1、TERT、AXIN1、

		CPAMD8、EPCAM、GSTP1、MEN1、NF2、PTEN、<br><br>SH3BP5、TIRAP
**A broad-spectrum panel genes**	**17**	AKT1 、ALK 、ARAF、 BCL2L11、BRAF、CYP2C19、DPYD 、EGFR 、ERBB2 、ESR1 、FGFR1 、FGFR2 、FGFR3 、GNA11 、GNAQ 、HRAS 、IDH1 、IDH2 、KIT 、KRAS 、MAP2K1 、MET 、MTOR 、NRAS 、NTRK1 、NTRK3、PDGFRA、PIK3CA 、RET 、TPMT 、UGT1A1
