# Supplementary material for: Mutational Profiling Detection in FNAC Samples of Different Types of Thyroid Neoplasms Using Targeted NGS
Source: Cancers (Basel). 2025 Jul 23;17(15):2429. doi: 10.3390/cancers17152429 (PMC12346461; doi:10.3390/cancers17152429)
Supplement: Supplementary file 1 [file cancers-17-02429-s001.zip › cancers-3720217 Supplementary Table S4.pdf]

Supplementary Table S4. Distribution of Thyroid Cancer Subtypes Across Different Gene Panels

		BT	LRN	PTC	FTC	PDTC& ATC	MTC
	**Numbers**	14	12	907	5	9	5
**18 Gene Panel**	**328**	4（28.57%）	4（33.33%）	326（35.94%）	0	0	0
**88 Gene Panel**	**607**	10（71.43%）	8（66.67%）	566（62.40%）	5（88.89%）	8（88.89%）	4（80.00%）
**Pan-Cancer Gene Panel**	**17**	0	0	15（16.54%）	0	1（11.11%）	1（20.00%）
