# Supplementary material for: Mutational Profiling Detection in FNAC Samples of Different Types of Thyroid Neoplasms Using Targeted NGS
Source: Cancers (Basel). 2025 Jul 23;17(15):2429. doi: 10.3390/cancers17152429 (PMC12346461; doi:10.3390/cancers17152429)
Supplement: Supplementary file 1 [file cancers-17-02429-s001.zip › cancers-3720217 Supplementary Table S5.pdf]

Supplementary Table S5: $BRAF^{V600E}$ Mutation Frequency, Lymph Node Metastasis, and Tumor Size by Mutation Burden

	SUM	$BRAF^{V600E}$	$BRAF^{V600E}$ /SUM（%）	LNM Rates（%）	Tumor size（mm）
**1**	510	446	87.45	58.27	10.96
**2**	216	187	86.57	58.88	10.98
**3**	107	97	90.65	47.62	12.51
**4**	63	53	84.13	60.32	12.65
**＞4**	27	18	66.67	75.00	19.24
